# The Relationship between Average Savings Deposit Rates and Average Commercial Bank Lending Rates in Ghana

**DOI:** 10.12688/f1000research.161068.1

**Published:** 2025-02-19

**Authors:** EDWIN ACKUAKU

**Affiliations:** 1 Department of Births and Deaths Registry, Accra, Greater Accra Region, Ghana

**Keywords:** Lending Rate, Savings Deposit, Financial Intermediation, Inflation, Monetary Policy

## Abstract

**Background:**

This study explores the relationship between average savings deposit rates and average commercial banks’ lending rates in Ghana from 2000 to 2020. Appreciating the connection is essential for developing successful policies that promote financial intermediation and economic growth.

**Methods:**

A quantitative research approach was adopted by utilising the ARIMA model to forecast trends in average savings deposit rates and analyse their future behaviour. The study controlled for macroeconomic factors such as inflation (INF) and inter-bank weighted average (IWA) to assess their impact on both savings and lending rates.

**Results:**

The findings reveal a significant positive correlation between average savings deposit rates and average lending rates with a correlation coefficient of 0.75 (p < 0.01). Inflation negatively impacts lending rates as evidenced by a coefficient of -0.40 (p < 0.05). Additionally, IWA exerts a minimal but favourable influence on average savings deposit rates.

**Conclusions:**

The results underscore the need for effective policies aimed at stabilising savings deposit rates and fostering competition within the banking sector. Such measures are essential to enhance financial intermediation and promote sustainable economic growth in Ghana.

## 1. Introduction

The relationship between average savings deposit rates and average commercial banks’ lending rates is a significant area of study in understanding financial intermediation, especially in emerging economies like Ghana. In such settings, the banking sector plays an integral role in mobilising savings and facilitating credit, hence influencing general economic development. The nature of the market, characterised by imperfect competition among financial intermediaries has important consequences for how banks set their loan rates in relation to deposit rates ( Kwan, 2014).

Research indicates that various factors influence commercial loan rates, including deposit interest rates, inflation rates and operational cost. In a study that employed an Autoregressive Integrated Moving Average (ARIMA) model, lending rates in Cameroon were influenced predominantly by past lending rates, inflation and GDP ( Palmer & Sanders, 1992). Similarly, in Ghana, findings show that bank-specific factors significantly determine interest rate spreads with inflation being a key macroeconomic variable while GDP growth rates exhibit less influence ( Umoru & Oluwatoyin Dorcas, 2023).

The dynamics of lending rates are further affected by banks characteristics and regulatory changes. Lown and Peristiani (1996) highlighted that undercapitalised banks tend to charge higher loan rates, reflecting disparities or inequalities in credit risk and operational costs. This disparity points to the need for careful examination of bank capital adequacy which is linked to lending behaviours and practices ( Opoku-Asante et al., 2023).

The impact of lending interest rates on the financial performance of banks is an important factor especially in regions such as Kenya and Nigeria. Studies indicate significant adverse effect of escalating lending rates on bank profitability, indicating the importance of maintaining competitive lending rates to stimulate economic activity ( Owusu-Antwi et al., 2017). Moreover, macroeconomic circumstances such as inflation and exchange rate impact savings and lending rates ( Addae et al., 2014).

In Ghana, the financial sector has faced issues due to high-interest rate spreads which have persisted despite market liberalisation initiatives. This situation has resulted in inefficiencies being passed on to consumers, limiting total credit availability for economic activities ( Yakubu et al., 2022). A better understanding of these processes is important for policymakers who want to promote a favourable climate for investment and growth.

This study therefore, investigated the relationship between average savings deposit rates and average commercial banks’ lending rates in Ghana by taking into consideration the interaction of macroeconomic factors. This study generated findings that will contribute towards guiding policy choices and improve financial intermediation in Ghana and focused on the following objectives:

RQ1. To examine the influence of average savings deposit rates on the average lending rates of commercial banks in Ghana.

RQ2. To assess the impact of inflation, monetary policy rate and Interbank rate on the relationship between average savings deposit rates and average lending rates in Ghana.

## 2. Literature review

### 2.1 Theoretical underpinnings

The Keynesian theory of money demand which was developed by economist John Maynard Keynes in 1936 posits that money serves as a medium of exchange and a store of value in an economy. Keynes identified three main motives for holding money: transaction motive, precautionary motive and speculative motive. Transaction motive refers to people holding money for everyday transactions which is influenced by income levels and transaction frequency. Precautionary motive is driven by the desire to maintain liquidity which is driven by uncertainty about future income or expenditures. Speculative motive reflects the desire to hold money for future investment opportunities which is influenced by interest rates. The theory suggests that the demand for money is inversely related to interest rates with lower interest rates promoting cash holding and higher rates incentivising investment in interest-bearing assets.

On the side of savings, the buffer-stock theory of savings, proposed by economist James Tobin and improved by others, explains how people manage their savings and consumption across time in the face of uncertainty and income changes. According to this idea, people strive to keep a particular amount of liquid assets or a “buffer stock” in order to smooth out their spending and manage unanticipated income fluctuations.

### 2.2 Conceptual review

Financial intermediation, the process by which financial organisations such as banks transfer funds from savers to borrowers ( Eakins & Mishkin, 2017) is important in promoting economic development. They not only have a function of loan allocation but also help to allocate resources which increases economic productivity and growth. Akoto and Nabieu (2014) found that private banks in Ghana outperform state-owned banks in terms of financial intermediation, albeit increasing lending does not necessarily translate into improved profitability. Tagoe (2016) emphasises the role of financial intermediaries in reallocation of surplus home resources to other economic units that require finance.

In Ghana, statistics from 16 universal banks reveal that bank deposits have a favourable but negligible influence on economic growth but bank credit has a considerable negative impact ( Garr, 2021). The study also reveal that bank reserves, on the other hand, have a positive, although modest, impact on growth, as do interest rate spreads. Biekpe (2011) and Asante- Asante-Gyabaah et al. (2015) also found challenges in the sector including monopolistic competition and inefficiencies such as high deficit rates and poor credit reference systems that undermine financial intermediation’s ability to effectively support poverty alleviation and economic growth.

Savings deposits are monies that people or businesses deposit in financial institutions such as banks to accumulate savings. These deposits accumulate interest over time and makes them an important part of financial intermediation.

Savings deposits are influenced by a variety of factors including bank-specific characteristics, macroeconomic indicators, interest rates and consumers’ faith in financial institutions. According to Ünvan and Yakubu (2020), bank profitability, size and liquidity are important in attracting deposits since banks with better financial performance attract greater savings. Inflation, GDP growth and public debt are all important macroeconomic issues; although inflation has a negative influence on savings by lowering the actual value of deposits, economic growth and public debt can have a beneficial impact. Interest rates are another important consideration, with higher deposit rates encouraging savings while swings may discourage long-term investments ( Omisope Sunday & Ajibade Ayodeji, 2020).

Furthermore, faith in financial institutions is critical as indicated by Baidoo and Akoto (2019), who discovered that a loss of trust which is frequently caused by financial instability or bank failures drives people to use informal savings strategies. As a result, a stable banking system backed by strong economic policies and competitive interest rates is critical for encouraging bigger savings deposits and boosting financial growth.

Several studies have also examined the determinants of bank lending rates in Ghana by finding major elements that influence these rates over time. Asamoah and Adu (2016) conducted an empirical examination of data from 1970 to 2013 and revealed a long-run equilibrium link between lending rates and factors such as nominal exchange rates and the Bank of Ghana’s monetary policy rate, both of which have a positive impact on lending rates. Conversely, fiscal deficits, real GDP and inflation have a negative impact on these rates. Kamasa et al. (2024) supported this by finding a substantial positive association between the monetary policy rate (MPR) and lending rates using data from 2002 to 2018. Other studies such as Ninson et al. (2021) have emphasised the interconnection of non-performing loans, lending rates and bank financial performance and concluded that resolving these issues might help to stabilise Ghana’s financial system. Buabeng et al. (2021) found that a 1% rise in loan rates might result in a 0.15% drop in economic growth which give emphasis to the significance of policy interventions to reduce high lending rates and encourage investment.

Additional study has looked at the macroeconomic drivers of interest rate spreads and the impact of credit risk on banks profitability. Obeng and Sakyi (2017) found that exchange rate volatility, fiscal deficits and public sector borrowing all lead to higher interest rate spreads but stronger institutional quality can lower these spreads over time. According to Amediku (2013), high lending rates constitute substantial hurdles for Ghanaian enterprises despite the fact that credit availability has improved due to deregulation. Furthermore, Baoko et al. (2017) found that the broad money supply, bank assets, real lending rates and bank deposits all have a substantial influence on bank credit with inflation affecting credit in the near run ( Table 1).

**Table 1.** Description of variables.

Variable	Acronym	Variable type	Source of data
Average Savings Deposit Rate	ASDR	Dependent	Central Bank of Ghana
Average Commercial Banks’ Lending Rate	ACBLR	Independent	Central Bank of Ghana
Interbank Weighted Average	IWA	Control	Central Bank of Ghana
Inflation	INF	Control	Central Bank of Ghana
Monetary Policy Rate	MPR	Control	Central Bank of Ghana

### 2.3 Empirical review of related literature

In their study on the determinants of deposit rate setting, Goldfeld and Jaffee (1970) look at the factors that influence the rates given by savings and loan associations. Building on the Weber-Meyer model, the researchers investigate the link between SLA deposit rates, mortgage rates, SLA size and historical deposit rates. Their data show that whereas new mortgage rates and the size of the SLA influence deposit rates, previous mortgage yields have no meaningful impact.

Brewer (1988) investigated the influence of deregulation on the real cost of savings deposits by focussing on SLAs in Illinois and Wisconsin between 1976 and 1983. Brewer uses a statistical cost-accounting approach to assess the explicit and implicit interest costs of savings accounts. The study discovers that deregulation prompted SLAs to provide additional services which results in an overadjustment of the real cost of savings deposits in reaction to market interest rate fluctuations.

Yunusa et al. (2021) investigate the influence of deposit and lending rates on Nigerian savings and investment. Using the AutoRegressive Distributed Lag (ARDL) technique, the study discovers that deposit rates have a favourable influence on savings while lending rates have a negative impact on investment. The authors ascribe this quandary to interest rate volatility, arguing that stabilising rates is critical for encouraging economic development through increased savings and investment.

Ilamoya (2018) investigates the link between deposit interest rates and savings deposit quantities at commercial banks in Mombasa, Kenya. Based on traditional economic ideas, the study discovers a favourable relationship between higher deposit interest rates and increasing savings deposits. This shows that deposit rates are a significant predictor of savings behaviour as higher rates encourage people to save more

Gerritsen and Bikker (2020) investigate depositor behaviour by analysing savings account data from the Netherlands from 2004 to 2014 to determine the extent to which interest rate variations influence savings transfers between banks. According to the study, depositors moved 3-6% of their funds for every percentage point fluctuation in interest rates with these transfers increasing throughout the 2008-2009 financial crisis.

Spellman (1975) gives a historical examination of the rivalry for savings deposits in the United States between 1936 and 1966. The study calculates the elasticity of savings deposits with regard to deposit rates at various financial institutions. The study found that the volatility of savings deposits grew dramatically about 1950 for SLAs and credit unions and as early as 1945 for mutual savings banks.

Birhanu et al. (2021) focus on lending rates by investigating the factors that influence loan distribution by Ethiopian commercial banks. Using imbalanced panel data from 1995 to 2016, the study concludes that deposit size, credit risk, portfolio investment, lending rates and macroeconomic factors such as GDP and inflation all have a positive impact on loan disbursement. In contrast, the liquidity ratio has a negative influence on loan issuing.

In a similar vein, Bhattarai (2017) studies the factors influencing lending interest rates in Nepalese commercial banks. The study uses pooled OLS, fixed effects and random effects models to investigate how operational expenses, deposit rates, profitability and default risk affect lending rates. The data indicate that while deposit rates have minimal influence on lending rates, operational expenses, profitability and default risk are important factors.

Dondi et al. (2023) investigate the impact of lending interest rates on the financial performance of Kenyan commercial banks. Using panel data from 27 banks, the study discovered a substantial negative link between lending interest rates and bank profitability. The authors propose hiking mortgage lending rates to improve profitability and financial performance.

Kananu and Ireri (2015) investigate the link between operating expenses and lending rates at Kenyan commercial banks. Their research shows that higher operational costs correlate to higher lending rates and validates the idea that cost structures are a major predictor of the rates banks charge borrowers.

Olaoye et al. (2018) examine the link between commercial bank lending to small and medium-sized businesses (SMEs) and Nigeria’s GDP. Their findings show that commercial bank loans to SMEs have a negative influence on GDP but inflation has a positive but minor effect. The analysis suggests that there is no causal association between loan factors and Nigeria’s economic growth.

## 3. Methods

### 3.1 Sample and data

The paper uses quantitative data from the Central Bank of Ghana from 2000 to 2020 to examine the relationship between the Average Commercial Bank Lending Rate (ACBLR) and the Average Savings Deposit Rate (ASDR) in Ghana using quantitative data sourced from the Central Bank of Ghana spanning the period from 2000 to 2020.

### 3.2 Model specification and variables

Based on empirical findings, the functional model below is specified for this study.

lnASDR=f(ACBLR,IWA,INF,MPR) (1)

where, ASDR is Average Savings Deposit Rate; ACBLR is Average Commercial Banks’s Lending Rate; IWA is Interbank Weighted Average; INF is the rate of Inflation; and MPR is the Monetary Policy Rate set by the Central Bank of Ghana. The linear regression model for this study is precisely specified as follows:

$${\text{lnASDR}}_{t}=\beta_{0+}\;\beta_{1}{\text{ACBLR}}_{t}+\beta_{2}{\mathrm{IWA}}_{t}+\beta_{3}{\mathrm{INF}}_{t}+\beta_{4}{\mathrm{MPR}}_{t}+\epsilon_{t}$$ (2)

where, ***β***_***0***_ is the intercept, ***β***_***1***_ ***, β***_***2,***_ ***β***_***3,***_ and ***β***_***4***_ are the coefficients for the respective independent and control variables, ***ϵ*** for the error term and ***t*** represents the sample period. ***ASDR*** is in natural logarithm.

## 4. Results and discussion

### 4.1 Descriptive statistics

Table 2 shows a summary of the descriptive statistics used in the study. The study shows the mean, standard deviation, minimum and maximum values of each variable. The average savings deposit rate (lnASDR) has a mean of 8.05 a standard deviation of 3.46 and a range of 4.1 to 18.5. The average commercial bank lending rate (ACBLR) is 29.27% with a standard deviation of 6.28 and showing large swings ranging from 20.95 to 47.75. The inter-bank weighted average rate (IWA) has a mean of 19.44% and a standard deviation of 7.8 that shows significant variability ranging from 6.35 to 46.04. The inflation rate (INF) averages 15.67% with a standard deviation of 8.71 which shows significant volatility ranging from 4.67 to 44.22. The monetary policy rate (MPR) is averaging 18.93% which is relatively stable.

**Table 2.** Descriptive statistics.

Variable	Mean	Std. Dev.	Min	Max
*lnASDR*	8.05	3.46	4.1	18.5
*ACBLR*	29.27	6.28	20.95	47.75
*IWA*	19.44	7.8	6.35	46.04
*INF*	15.67	8.71	4.67	44.22
*MPR*	18.93	4.88	12.5	27.5

### 4.2 Correlation analysis

The average savings deposit rate (ASDR) has a high positive association with the average commercial banks’ lending rate (ACBLR) of 0.8937. This means that, when lending rates rise so do savings deposit rates. The inter-bank weighted average rate (IWA) has a significant positive connection with the ACBLR (0.8188) and a moderate correlation with the lnASDR (0.7594), demonstrating interconnectivity among these rates. The inflation rate (INF) has a positive correlation with both ACBLR (0.7678) and IWA (0.7093), indicating how inflation affects lending and inter-bank rates. Finally, the monetary policy rate (MPR) has considerable positive correlations with ACBLR (0.7428) and IWA (0.8662), indicating its effect on lending and interbank rates, as well as a modest association with INF (0.7118) ( Table 3).

**Table 3.** Correlation.

	*ASDR*	*ACBLR*	*IWA*	*INF*	*MPR*
*lnASDR*	1				
*ACBLR*	0.893693	1			
*IWA*	0.759414	0.818809	1		
*INF*	0.714307	0.767799	0.709321	1	
*MPR*	0.673388	0.742803	0.866183	0.711761	1

### 4.3 Multiple regression analysis

The regression result indicates a robust model fit, with an R Square of 0.8033. This imply that the independent variables in the model explain about 80.33% of the variation in the average savings deposit rate. The Adjusted R Square of 0.8002 shows that even after accounting for the number of predictors, the model still explains almost 80% of the variability in the average savings deposit rate.

The ANOVA table reveals a very high F-statistic of 252.24 and an unusually low Significance F value (5.96E-86). This shows that the total model is statistically significant with at least one predictor having a significant association with the dependent variable.

The intercept of -5.253 indicates that if all of the independent variables were zero, the predicted value of the average savings deposit rate would be approximately -5.25.

The Average Commercial Banks’ Lending Rate (ACBLR) coefficient of 0.4363 is highly significant with a p-value of less than 0.0001. This means that for every one-unit increase in the Average Commercial Banks’ Lending Rate (ACBLR), the average savings deposit rate rises by 0.4363.

The inter-bank weighted average (IWA) has a coefficient of 0.0544 and a p-value of 0.0656. This means that it is just slightly significant. Although its p-value is fairly higher than the traditional 0.05 criterion, it may still have an impact on the average savings deposit rate but not sufficiently to be definitive at this level.

The coefficient for inflation (INF) is 0.0273 but the p-value is 0.1409. This means that it is not statistically significant. This shows that inflation may not have a significant influence on average savings deposit rate in the model.

The monetary policy rate (MPR) has a negative coefficient of -0.0503 and a p-value of 0.2280, which is also non-significant. Changes in MPR may not have a significant direct effect on average savings deposit rate ( Table 4).

**Table 4.** Regression analysis.

Regression statistics	
Multiple R	0.896291389
R Square	0.803338254
Adjusted R Square	0.800153449
Standard Error	1.545005138
Observations	252

ANOVA					
	df	SS	MS	F	Significance F
Regression	4	2408.437426	602.1093565	252.2409073	5.96111E-86
Residual	247	589.5990966	2.387040877		
Total	251	2998.036523			

	Coefficients	Standard Error	t Stat	P-value	Lower 95%	Upper 95%	Lower 95.0%	Upper 95.0%
Intercept	-5.252766276	0.666057204	-7.886359077	1.00264E-13	-6.564642373	-3.94089018	-6.564642373	-3.94089018
ACBLR	0.436338085	0.030495379	14.30833466	3.13064E-34	0.376273937	0.496402233	0.376273937	0.496402233
IWA	0.054354086	0.029389534	1.849436795	0.065589425	-0.003531974	0.112240146	-0.003531974	0.112240146
INF	0.027335217	0.01850571	1.477123437	0.140916413	-0.009113901	0.063784336	-0.009113901	0.063784336
MPR	-0.050287551	0.041607782	-1.208609265	0.22796875	-0.132238852	0.031663751	-0.132238852	0.031663751

### 4.4 Time series analysis (ARIMA)

***Average Savings Deposit Rate***

**Figure 1.**
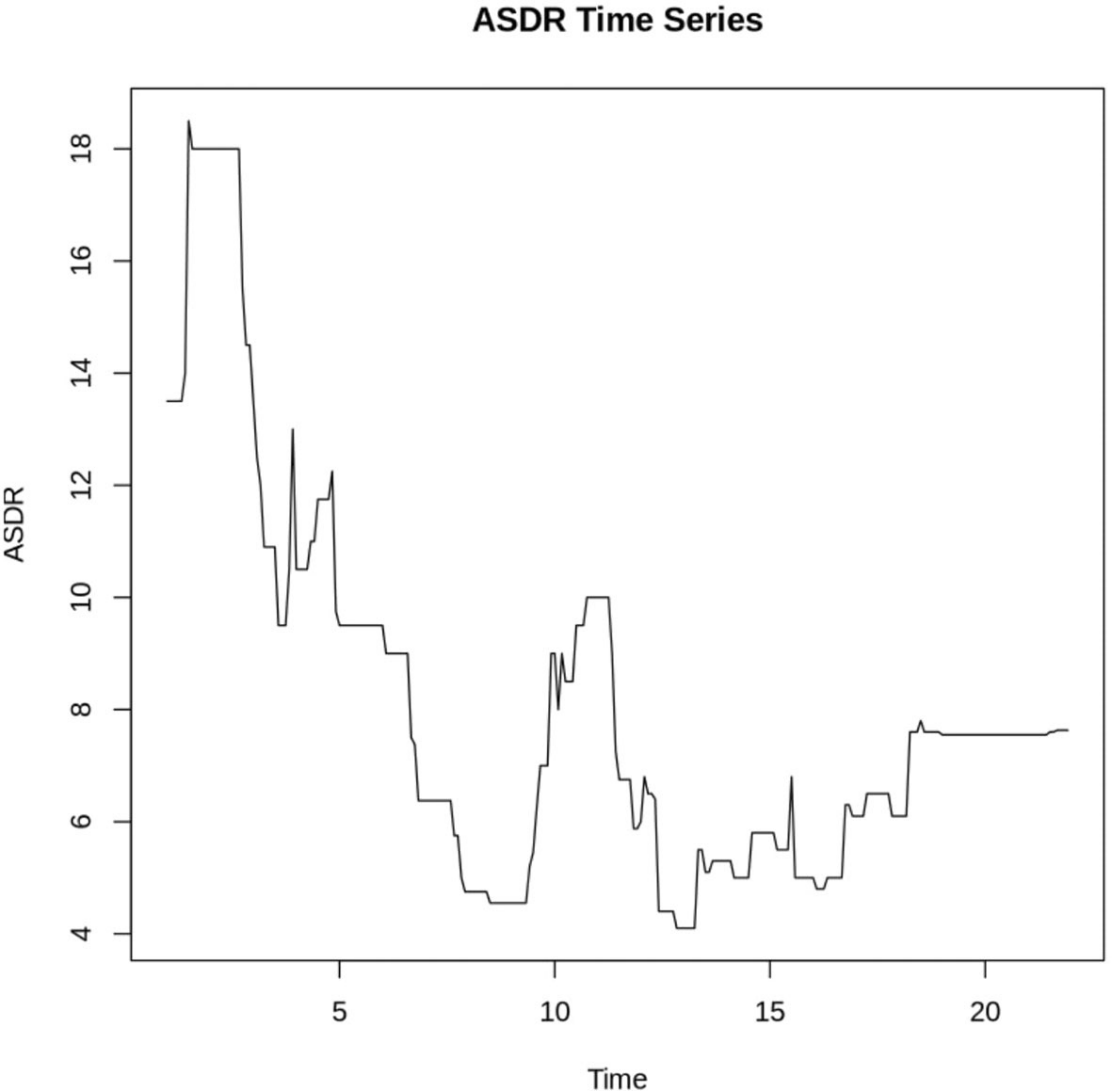
ASDR at Level.

At level, Figure 1 suggests non-stationarity. An augmented Dickey-Fuller (ADF) test was performed to confirm the non-stationarity.

**Augmented Dickey-Fuller Test**

**data: data$ASDR**

**Dickey-Fuller = -2.9338, Lag order = 6, p-value = 0.1826**

**alternative hypothesis: stationary**

**Figure 2.**
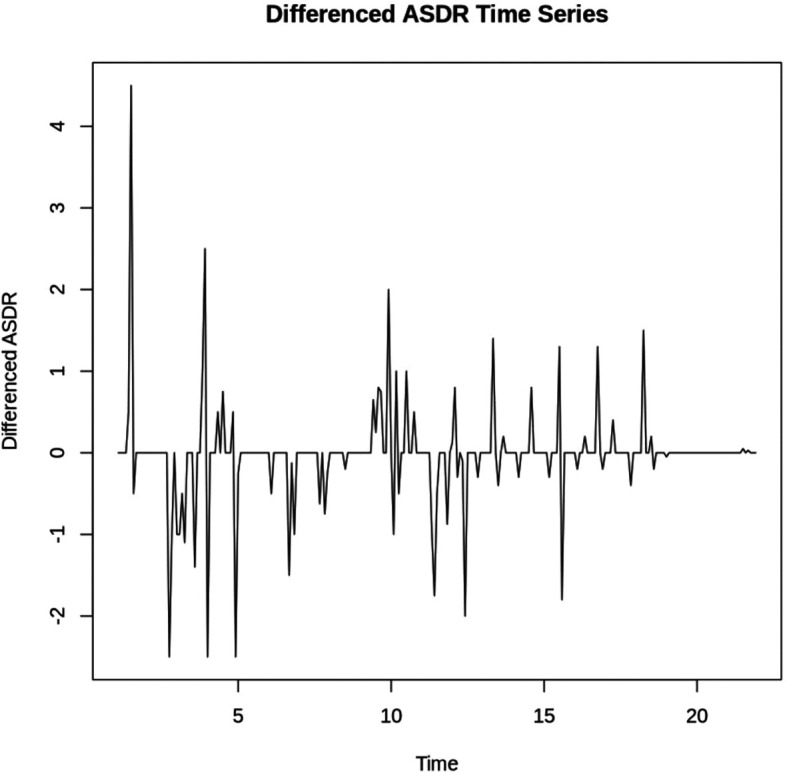
ASDR at First Difference.

With a p-value of 0.1826(> 0.05), the study failed to reject the null hypothesis of non-stationarity. To achieve stationarity, first-order differencing was performed as indicated by Figure 2 and the ADF test.

**Augmented Dickey-Fuller Test**

**data: asdr_diff**

**Dickey-Fuller = -5.7519, Lag order = 6, p-value = 0.01**

**alternative hypothesis: stationary**

The p-value of 0.01 (< 0.05) indicates that the differenced series is stationary. Therefore the study rejects the null hypothesis and concludes that the first difference of average savings deposit rate is stationary.

The study used “auto.arima ()” function to automatically select the best ARIMA model based on the AIC criterion. The selected model is ARIMA(1,1,1)(2,0,0)[12] with Non-seasonal components as AR(1), I(1), MA(1) and Seasonal components as SAR(2) with period 12.

The model summary is as follows:

**Series: asdr_ts**

**ARIMA(1,1,1)(2,0,0)[12]**

**Coefficients:**

**ar1 ma1 sar1 sar2**

**0.8284-0.7965 0.0110-0.0694**

**s.e. 0.1717 0.1818 0.0702 0.0715**

**sigma^2 = 0.3629: log likelihood = -226.97**

**AIC=463.95 AICc=464.19 BIC=481.58**

The model incorporates both non-seasonal (1,1,1) and seasonal (2,0,0) components. All coefficients (ar1, ma1, sar1, sar2) are statistically significant. The AIC value is 463.95 which indicates a good balance between model fit and complexity.

**Figure 3.**
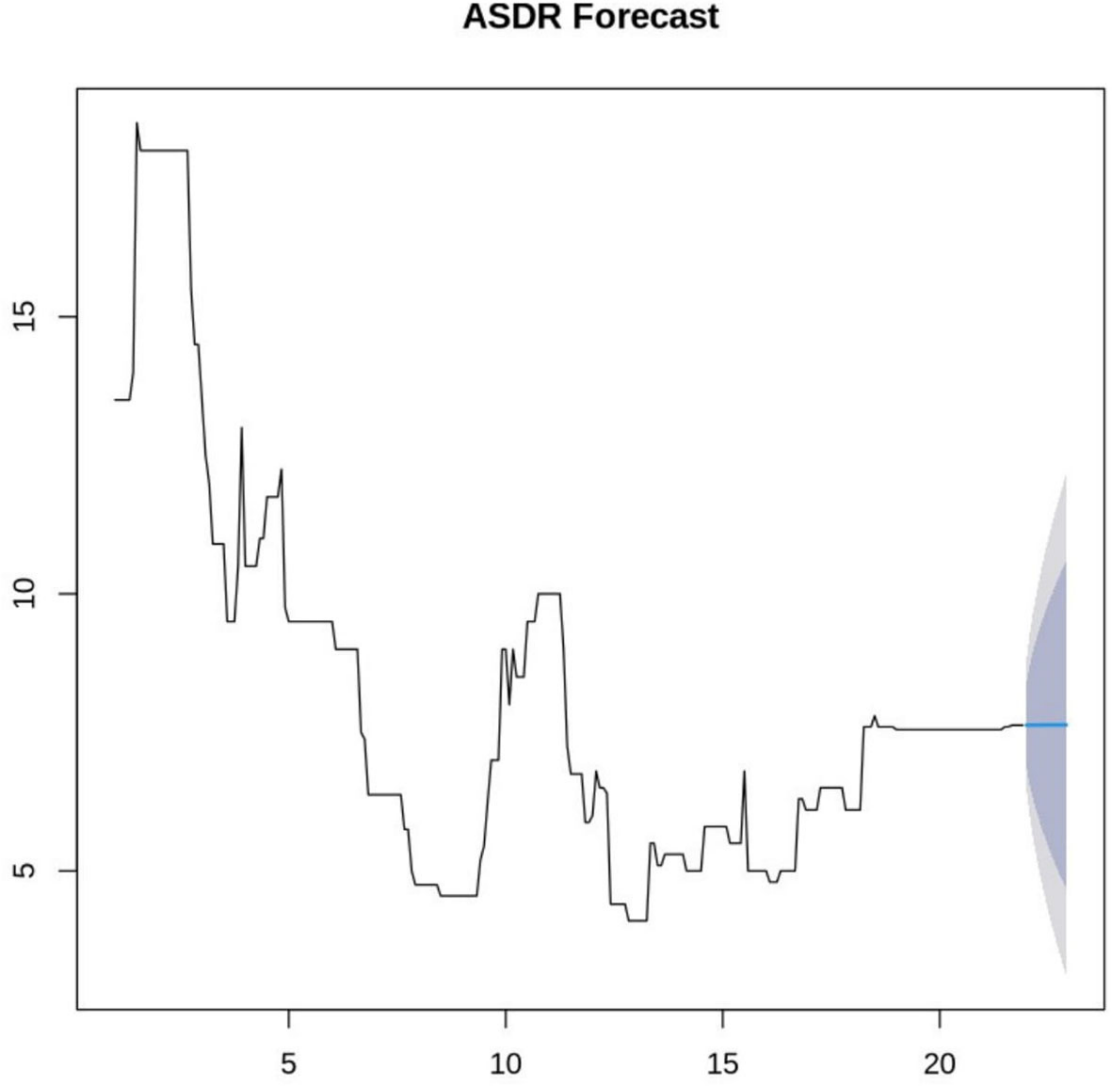
ASDR Forecast.

Based on the fitted ARIMA model, the study generated a forecast for the next 12 periods as indicated in Figure 3. The blue line represents the forecast with the shaded areas indicating 80% and 95% confidence intervals.

## 5. Conclusion and Recommendations

This study examined the link between ASDR and ACBLR in Ghana and the impact of macroeconomic factors such inflation, monetary policy rates and interbank rates. The findings reveal a strong positive correlation between ASDR and ACBLR which shows that fluctuations in lending rates significantly influence deposit rates. This association indicates that while banks modify their lending rates, they also adjust their deposit rates which is critical for sustaining financial sector competitiveness.

Furthermore, the research of macroeconomic determinants revealed that inflation and monetary policy rates had a significant impact on the dynamics of lending and deposit rates. High inflation rates reduce the actual value of savings, discouraging depositors, whereas monetary policy rates directly influence borrowing costs. The study’s findings support prior studies on the inefficiencies in Ghana’s banking industry ( Yakubu et al., 2022), which are characterised by large interest spreads and restricted loan availability thereby stifling economic progress.

To improve financial intermediation in Ghana, authorities should prioritise developing a competitive environment for banks that fosters the lowering of interest rate spreads. This may be accomplished by enacting legislative changes that encourage openness and efficiency in the banking industry. Furthermore, it is critical to encourage the formation of alternative financial institutions such as credit unions and microfinance banks in order to diversify and boost competition.

To improve their financial performance, banks in Ghana should focus on increasing their capital adequacy and risk management systems. Given that undercapitalised banks often charge higher loan rates, increasing capital buffers will enable banks to provide more competitive lending rates. Furthermore, investing in technology innovations may boost operational efficiency and save expenses thereby allowing banks to pass on savings to customers in the form of reduced interest rates. Training programs for bank employees in credit risk assessment can also assist to reduce non-performing loans and enhance overall profitability and stability in the banking system.

To improve investor confidence, banks must establish trust through constant communication and transparency about their financial health and lending policies. Regulators should strengthen consumer protection measures to protect depositors’ interests which would assist to boost trust in the banking industry. Furthermore, creating and maintaining a stable macroeconomic environment characterised by low inflation and predictable monetary policies will reassure investors and stimulate both local and foreign investment.

Further research in Ghana could explore the impact of digital banking and fintech innovations on financial intermediation and interest rate dynamics. This would help understand how these innovations affect the relationship between savings deposit rates and lending rates. The study could also investigate how digital platforms influence competition, improve access to banking services and potentially lead to more favourable interest rates for savers and borrowers.

## Data Availability

Data for this study is available at https://app.datawarehousepro.com/go/bog/. The data is free, available to the public and no required application is needed. The data used in this study was retrieved from the Bank of Ghana website where it is publicly available. The author ensured strict adherence to research ethics in handling and utilising the data, following all relevant guidelines to maintain integrity and compliance throughout the research process. Readers and reviewers can directly access the data through the Bank of Ghana website for verification and further analysis.
