# Supplementary material for: The Relationship between Average Savings Deposit Rates and Average Commercial Bank Lending Rates in Ghana
Source: F1000Res. 2025 Feb 19;14:222. [Version 1] doi: 10.12688/f1000research.161068.1 (PMC12314481; doi:10.12688/f1000research.161068.1)
Supplement: Peer review history

# Reviewer report: Reviewer response for version 1

**DOI:** 10.5256/f1000research.177051.r398787

**Published:** 2025-12-03

**Authors:** Patrick Gudda

**Affiliations:** 1 Maasai Mara University, Narok, Narok County, Kenya

**The Relationship between Average Savings Deposit Rates and Average Commercial Bank Lending Rates in Ghana**

**General Overview**

 The study explores an important subject within financial economics, examining the relationship between deposit and lending rates using data from the Central Bank of Ghana spanning 2000 -2020. The use of ARIMA for forecasting and multiple regression for explanatory analysis is statistically sound and adds analytical depth. The topic is policy relevant, especially for developing economies like Ghana.

 However, for indexing several substantive and editorial revisions are needed.

**1. Reframe Focus to Interest Rate Spread**

**Section: Abstract (Page 1)**

 “The findings reveal a significant positive correlation between average savings deposit rates and average lending rates…” - This statement emphasizes a known relationship rather than providing new insight.

**Suggestion:** Replace with an emphasis on interest rate spreads; the difference between the two rates and their implications on financial intermediation and credit accessibility in Ghana.

**2. Explain the Negative Inflation-Lending Rate Relationship**

**Section:** **Results and Discussion → Regression Analysis (Page 7)**

 “The coefficient for inflation (INF) is 0.0273 but the p-value is 0.1409. This shows that inflation may not have a significant influence...”

- **Conclusion (Page 10):**

“...inflation and monetary policy rates had a significant impact on the dynamics of lending and deposit rates...” - The discussion of inflation’s role is inconsistent and lacks theoretical grounding. Inflation’s impact appears both significant and insignificant.

**Suggestion:** Expand with theoretical perspectives: monetarist vs. Keynesian, Ghana's inflationary history, and central bank policy context.

**3. Improve Language and Academic Style**

**Section(s) Throughout Document:**

- **Abstract (Page 1):** “Appreciating the connection is essential…”
- **Introduction (Page 2):** “This disparity points to the need for careful examination…”
  “This study therefore, investigated…”

**Suggestion: **Avoid informal or colloquial phrases that lower academic tone. Replace with formal academic expressions such as “Understanding this relationship is vital for formulating monetary policy…” or “Accordingly, the study investigates…”

**4. Clarify Methodology and Causality**

**Section:** **Methods – Model Specification (Page 5):**

 “lnASDR = f (ACBLR, IWA, INF, MPR)”

 The dependent variable is savings deposit rate, yet the main interest is in lending rates. This raises concerns about **causality direction**.

**Suggestion: **Justify choice of dependent variable, discuss potential endogeneity, and consider Granger causality testing or Vector Autoregression (VAR) approach.

**5. Reduce Redundancy and Repetition**

**Section:** **Introduction & Literature Review (Pages 2–4):**

 Many studies referenced in both sections, Addae et al. (2014), Yakubu et al. (2022), Owusu-Antwi et al. (2017).

**Suggestion:** Merge repetitive discussions and focus literature review on synthesizing themes rather than listing studies.

**6. Update and Narrow Literature Review**

**Section:** **Literature & Empirical Review (Pages 4–5):**

 Spellman (1975), Goldfeld & Jaffee (1970), Brewer (1988)

**Suggestion:** Remove or minimize older references and replace with studies post-2010 focusing on Africa, West Africa, or developing economies with similar financial structures.

**7. Strengthen Policy Recommendations**

**Section:** **Conclusion and Recommendations (Page 10):**

 “To improve financial intermediation in Ghana, authorities should prioritize developing a competitive environment…” - Policy proposals are generic and lack concrete links to empirical findings.

**Suggestion:** Suggest precise interventions: interest rate cap reforms, promotion of fintech lending platforms, capital adequacy monitoring, inflation targeting regimes.

**8. Clarify Role of ARIMA Forecast**

**Section:** **Time Series Analysis – ARIMA (Pages 8–9)**

 ARIMA forecasting is included but its relevance to policy or the regression model is unclear.

**Suggestion:** Clearly state whether the forecasted ASDR informs strategic planning or future deposit behavior under different lending regimes.

**Reviewer’s Remarks**

 While this study presents a relevant and empirically grounded exploration of the relationship between average savings deposit rates and lending rates in Ghana, it currently falls short of the analytical depth and academic rigor expected for publication in a high-quality research outlet. The author is commended for the effort in compiling longitudinal data and applying robust statistical tools such as regression and ARIMA models. However, substantial revisions are required to improve conceptual framing, clarify causal direction, strengthen theoretical alignment, refine language, and expand policy implications.

 By addressing the identified issues, especially the need to focus on interest rate spreads, reconcile inflation dynamics, and adopt a more scholarly tone, the manuscript will be significantly enhanced in both quality and impact. The topic remains of high relevance to scholars, policymakers, and practitioners in finance and development economics.

**Recommendation:** Major revisions required

Is the work clearly and accurately presented and does it cite the current literature?

Partly

If applicable, is the statistical analysis and its interpretation appropriate?

Partly

Are all the source data underlying the results available to ensure full reproducibility?

Yes

Is the study design appropriate and is the work technically sound?

Partly

Are the conclusions drawn adequately supported by the results?

Yes

Are sufficient details of methods and analysis provided to allow replication by others?

Yes

Reviewer Expertise:

Micro finance, innovation, entrepreneurship

I confirm that I have read this submission and believe that I have an appropriate level of expertise to confirm that it is of an acceptable scientific standard, however I have significant reservations, as outlined above.

# Reviewer report: Reviewer response for version 1

**DOI:** 10.5256/f1000research.177051.r398791

**Published:** 2025-07-31

**Authors:** Sabeha Barzan Farhod

**Affiliations:** 1 University of Baghdad, Baghdad, Baghdad Governorate, Iraq

The article discusses an important financial topic: examining the relationship between interest rates on savings accounts and interest rates on borrowing at the Central Bank of Ghana. The researcher used statistical equations such as multiple correlation, Multiple regression analysis,   standard deviation and arrived at reliable results that can be used in making economic decisions. The following observations represent the reasons for answering the questions:

1. Some of the references are outdated, dating back to 1970, 1975, 1988, 1992, 1996, 2013, 2014, 2016, and 2017. The researcher should rely on more recent references that address the topic.
2. The theoretical explanation and the review of the verses did not fully and comprehensively explain the title's terms. The researcher should explain the research terms broadly and comprehensively so that it can be used by researchers and academics.
3. The researcher did not include the data he depended on, to apply the statistical equations, and the link was not publicly available. The researcher must attach all financial data .

Is the work clearly and accurately presented and does it cite the current literature?

Partly

If applicable, is the statistical analysis and its interpretation appropriate?

Yes

Are all the source data underlying the results available to ensure full reproducibility?

No

Is the study design appropriate and is the work technically sound?

Yes

Are the conclusions drawn adequately supported by the results?

Yes

Are sufficient details of methods and analysis provided to allow replication by others?

Yes

Reviewer Expertise:

Financial Accounting, Auditing , Financial Analysis, Managerial and Cost Accounting, IFRS , International  Auditing Standards, International Accounting Standards ,

I confirm that I have read this submission and believe that I have an appropriate level of expertise to confirm that it is of an acceptable scientific standard, however I have significant reservations, as outlined above.

## References

[1] : Analysis of the Relationship between Credit Risk and Bank Performance of Some Commercial Banks in Ghana. *International Journal of Business and Management Research* .2020;8(2) : 10.37391/IJBMR.080202 34-51 10.37391/IJBMR.080202

[2] : The Efficiency of Domestic Savings and Its Impact on The Average Per Capita GDP In Iraq For the Period (2003-2022). *Journal of Economics and Administrative Sciences* .2024;30(141) : 10.33095/eenvvg64 385-405 10.33095/eenvvg64

[3] : Determinants of bank lending rates: Empirical evidence from conventional retail banks in Bahrain. *Banks and Bank Systems* .2022;17(4) : 10.21511/bbs.17(4).2022.12 140-153 10.21511/bbs.17(4).2022.12

[4] : SAVINGS AND INCOME RELATIONSHIPS AMONG HOUSEHOLDS: A REVIEW OF THE LITERATURE. *Agricultural Social Economic Journal* .2018;18(3) : 10.21776/ub.agrise.2018.018.3.6 133-143 10.21776/ub.agrise.2018.018.3.6

# Reviewer report: Reviewer response for version 1

**DOI:** 10.5256/f1000research.177051.r380633

**Published:** 2025-07-28

**Authors:** Dariusz Prokopowicz

**Affiliations:** 1 Cardinal Stefan Wyszyński University in Warsaw, Warsaw, Poland

The reviewed article titled *“The Relationship between Average Savings Deposit Rates and Average Commercial Bank Lending Rates in Ghana”* deserves a positive assessment due to its comprehensive and thorough approach to examining the relationship between deposit and lending interest rates in the context of macroeconomic conditions. The authors effectively identify a strong positive correlation between ASDR and ACBLR, which provides valuable insights for banking and macroeconomic policy in Ghana. Particularly noteworthy is the analysis of the impact of inflation and monetary policy interest rates on the behavior of the banking sector. The article also stands out for its practical approach, offering concrete recommendations for improving the efficiency and competitiveness of banks, developing alternative financial institutions, and building trust in the financial sector. An additional strength of the publication is its indication of future research directions, including the impact of digital banking and fintech innovations on the financial system, making the article highly relevant and significant for the advancement of economic sciences and public policy.

Is the work clearly and accurately presented and does it cite the current literature?

Yes

If applicable, is the statistical analysis and its interpretation appropriate?

Yes

Are all the source data underlying the results available to ensure full reproducibility?

Yes

Is the study design appropriate and is the work technically sound?

Yes

Are the conclusions drawn adequately supported by the results?

Yes

Are sufficient details of methods and analysis provided to allow replication by others?

Yes

Reviewer Expertise:

Macroeconomics, economic policy, banking and finance, credit risk management. In banking: central banking, monetary policy, etc.

I confirm that I have read this submission and believe that I have an appropriate level of expertise to confirm that it is of an acceptable scientific standard.

## References

[1] : THE POSTCOVID RISE IN INFLATION: COINCIDENCE OR THE RESULT OF MISGUIDED, EXCESSIVELY INTERVENTIONIST AND MONETARIST ECONOMIC POLICIES. *International Journal of New Economics and Social Sciences* .2022;16(2) : 10.5604/01.3001.0016.3409 105-148 10.5604/01.3001.0016.3409

# Reviewer report: Reviewer response for version 1

**DOI:** 10.5256/f1000research.177051.r378173

**Published:** 2025-06-20

**Authors:** Muhammad Ayub Mehar

**Affiliations:** 1 Iqra University Karachi, Karachi, Pakistan

This study establishes a relationship between two interest rates: interest on banks’ deposits, and interest on lending from commercial banks. The estimations are based on the data from commercial banks in Ghana from 2000 to 2020. The ARIMA model was applied to forecast the interest rate trends, while some macroeconomic factors including inflation and inter-bank weighted average have been included as controlled variables. The significant positive correlation between interest on deposits and interest on lending was explored, while inflation affects the interest on lending negatively.

 The ANOVA table shows a high F-statistic of 252 and an inordinate low significance level of F value, which shows statistical significance of the model as whole with at least one predictor having a significant association with the dependent variable.

 Despite its appropriate statistical methodology, and an interesting area of research which may be helpful for policymakers and academicians, I found some shortcomings:

  

1. First of all, it is a published paper, and available for reading, citation, and review. I suggest to consider also the suggestions and comments described by the readers.
2. The most important concern belongs to the testing of the correlation between the interest rate on deposits and interest rates on lending. These rates are calculated through weighted averages. Because these rates vary from bank to bank, or based on the types and purpose of lending and the risk associated with the lending etc. The important thing is that these two rates are always correlated. It is an obvious relation. A bank lends the money at a higher rate than the interest on deposits. The important is the "Interest rate spread", which is the difference between the two rates of interest. I strongly suggest that the authors must discuss the implications of interest rate spread, and mention if there are any regulatory or policy requirements to control the interest rate spread.
3. The negative relation between interest on lending and inflation is also questionable. The authors should explain the reasons. The monetarists' side explains the effectiveness of monetary policy in terms of its effects on growth. If the monetary policy is applied to reduce the rate of inflation, then the monetary authority (central bank) announces a higher policy rate. Sometimes this higher interest rate is determined based on expected inflation shortly. However, another view (usually from the lenders' side) is that the higher interest rate creates more inflation. I suggest that the author must discuss these theoretical implications, particularly in the context of Ghana.
4. The study is not good in academic writing, for instance, to say "Appreciating the connection is essential for developing successful policies that promote financial intermediation and economic growth" is not an academic way to present the context. The role of banks as an intermediary institution should be explained properly. There are several other mistakes in writing.

Otherwise, it is a good study, which can be considered for indexing after addressing the above-mentioned issues.

Is the work clearly and accurately presented and does it cite the current literature?

Partly

If applicable, is the statistical analysis and its interpretation appropriate?

Yes

Are all the source data underlying the results available to ensure full reproducibility?

Yes

Is the study design appropriate and is the work technically sound?

Yes

Are the conclusions drawn adequately supported by the results?

Partly

Are sufficient details of methods and analysis provided to allow replication by others?

Yes

Reviewer Expertise:

Corporate finance, Monetary economics.

I confirm that I have read this submission and believe that I have an appropriate level of expertise to confirm that it is of an acceptable scientific standard, however I have significant reservations, as outlined above.
